# Contemporary local anaesthetic-associated adverse events and mortality: a pharmacovigilance analysis of a US reporting system

**DOI:** 10.1016/j.bja.2025.06.044

**Published:** 2025-08-27

**Authors:** Michael R. Fettiplace, Guy Weinberg, Christopher Chiang, Heather C. Nixon, Marina Gitman

**Affiliations:** 1 Department of Anesthesiology, University of Illinois at Chicago, Chicago, IL, USA; 2 Department of Anesthesiology, University of Miami, Miami, FL, USA

**Keywords:** bupivacaine, lidocaine, local anesthetic systemic toxicity, mortality, pharmacovigilance, ropivacaine

## Abstract

**Background:**

One hundred years after the American Medical Association’s report on death from local anaesthetics, their contemporary lethality is not well characterised.

**Methods:**

We investigated local anaesthetic-associated deaths reported to the US Food and Drug Administration Adverse Event Reporting System from 1968 to 2023, comparing adverse events from local anaesthetics with adverse events from other drugs using the reporting odds ratio (ROR). We assessed the impact of professional society practice advisories about systemic toxicity on reporting of mortality from long-acting local anaesthetics and lidocaine. Finally, we extracted individual citations and conducted an exploratory analysis on cases of death.

**Results:**

There were 22 050 total adverse events from local anaesthetics and 1473 reports of death. Compared with other agents, reports of death were lower for amide (ROR, 0.4; 95% confidence interval [CI], 0.37–0.42) and ester (ROR, 0.12; 95% CI, 0.09–0.16) local anaesthetics. Death from lidocaine was reported most frequently. After practice advisories, reporting of death from long-acting local anaesthetics decreased (ROR, 0.61; 95% CI, 0.46–0.83) compared with the previous 10 yr, whereas reporting of death from lidocaine remained unchanged (ROR, 0.95; 95% CI, 0.78–1.16). Reporting of death from lidocaine was similar to other agents over the past 5 yr (ROR, 0.95; 95% CI, 0.83–1.1). Citations identified high-dose lidocaine, nerve catheters, and high neuraxial anaesthesia as the primary contributors to death since 2010.

**Conclusions:**

Reporting of mortality from long-acting local anaesthetics decreased in the past decade but remained unchanged for lidocaine. The potential lethality of this ubiquitous local anaesthetic deserves further study.

**Editor’s key points**

• Local anaesthetic-associated deaths reported to the US Food and Drug Administration Adverse Event Reporting System from 1968 to 2023 were compared with adverse events from other drugs.
• Reports of death were lower for amide and ester local anaesthetics than for other agents reported, with death from lidocaine reported most frequently.
• Reporting of death from long-acting local anaesthetics decreased after the introduction of practice guidelines compared with the previous 10 yr, whereas reporting of death from lidocaine remained unchanged.
• Deaths from lidocaine since 2010 were associated with use of high-dose lidocaine, nerve catheters, and high neuraxial anaesthesia.
• Whereas reporting of mortality from long-acting local anaesthetics decreased in the last decade, it remained unchanged for lidocaine. The reasons and mitigation strategies require further study.

Local anaesthetics (LAs) are ubiquitous in modern medicine, and they are used frequently for procedural pain control. In 1924, when drugs such as procaine (i.e. Novacaine) were still new, Mayer communicated on behalf of the American Medical Association an investigation of 43 deaths associated with LAs, along with recommendations intended to reduce mortality.^1^ A century later, we have estimates of the rates of LA systemic toxicity (LAST),^2^ but no more clarity about the risk of mortality based on type of LA. Because of its greater affinity for cardiac sodium channels and its history of causing intractable LAST, classical teaching identifies bupivacaine as the most dangerous LA. Additional research has reinforced the danger of bupivacaine compared with ropivacaine based on studies in cardiomyocytes.^3^ However, in the epoch of lipid emulsion treatment for LAST, ultrasound-guided blocks, large-volume ropivacaine infusions for truncal blocks, and professional society guidance on the treatment of LAST, what are the contemporary risks of bupivacaine-associated mortality?

To assess the relative risk of death from LAs, we queried the US Food and Drug Administration (FDA) Adverse Event Reporting System (FAERS) Public Dashboard^4^ to assess both relative reporting of mortality and changes in mortality since 2010, a year associated with treatment recommendations from multiple professional societies.^5^^,^^6^ We also reviewed the referenced literature in the FAERS database cited since 2010 to identify causes of death. We hypothesised that relative reporting of death from long-acting LAs (ropivacaine, bupivacaine, and levobupivacaine) has decreased since 2010.

## Methods

This analysis was classified as non-human subject research (University of Illinois at Chicago institutional review board: STUDY2023-1546).

### Data source

We conducted a pharmacovigilance case-non-case disproportionality analysis to investigate the relative mortality from LAs as reported to the FAERS dashboard. Cases were adverse events (AEs) related to LAs, and controls were AEs from other drugs. For cases, the FAERS dashboard^4^ was queried on April 3, 2024, for amide and ester LAs including articaine, benzocaine, bupivacaine, chloroprocaine, etidocaine, levobupivacaine, lidocaine, mepivacaine, prilocaine, procaine, proparacaine, ropivacaine, and tetracaine. Full search terms are found in the Supplementary material. We downloaded all records labelled as ‘serious’, removed duplicates, and separated records with an outcome of ‘died’. We only included records where LAs were named in isolation from other drugs in the ‘Suspect Product Active Ingredients’, except with other LAs or epinephrine. Records with other adjuvants or other drugs were removed; this included records that noted dextrose, sodium chloride, hyaluronidase, fentanyl, hydromorphone, methylprednisolone, morphine, propofol, prednisone, gabapentin, rocuronium, clonidine, dexamethasone, methadone, or tramadol. We extracted details on age, weight, and sex, and analysed for descriptive statistics. For controls, we queried total number of deaths and total number of reports from 1968 to 2023, tabulated records labelled as ‘serious’, and separated records with an outcome of ‘died’. For reporting odds ratio (ROR) calculations, we removed all LA-associated reports or deaths. Our outcomes of interest were rate of AEs with an outcome of ‘died’, and ROR of death from LA compared with death from other drugs in the database.^7^^,^^8^ We also compared ROR of death over multiple epochs. The ROR compares reporting of an AE of interest (i.e. death) with all other AEs, relative to the reporting for other drugs in the FAERS database. It is used as a standard measure in pharmacovigilance studies.^9^ Other drugs in the database include everything from sugammadex^7^ to immune checkpoint inhibitors,^10^ and conventional pharmacovigilance studies assess the whole database as the control comparator.

### Statistical analyses

We assessed the number of AEs with an outcome of ‘died’ associated with each LA or group of LAs as ${\text{AE}}_{\text{death}}=\frac{\text{AE}\;\text{with}\;\text{outcome}\;\text{of}\;‘\text{died}’\;}{\text{Total}\;\text{number}\;\text{of}\;\text{AEs}}$ for individual years and ranges of years (i.e. 1968–2023 and 2019–2023). For the AE_death_ rate, we calculated the standard error of the mean (sem) for mortality using the normal approximation of the binomial calculator: sem=x∗(N−x)N3.

The ROR of death from an LA was calculated as ${\text{ROR}}_{\text{death}\;\text{from}\;\text{LA}}=\left(\frac{\#\;\text{died}\;\text{from}\;\text{LA}}{\#\;\text{survived}\;\text{from}\;\text{LA}}/\frac{\#\;\text{died}\;\text{from}\;\text{other}\;\text{agents}}{\#\;\text{survived}\;\text{from}\;\text{other}\;\text{agents}}\right)$. An ROR with a confidence interval that does not cross 1 is considered significant. We calculated the ROR for both the overall period from 1968 to 2023 and for the most recent 5 yr of reporting (2019–2023) for each individual LA and for LAs grouped by class (amide or ester). ROR of death from amide-linked LAs was compared with ester-linked LAs.

To assess the impact of the 2010 practice advisory, we evaluated the ROR of death during the Workgoup Epoch (2011–2023) relative to the Pre-Workgroup Epoch (2001–2010) as ${\text{ROR}}_{\text{Workgroup}}=\left(\frac{\#\;\text{died}\;\text{from}\;\text{LA}\;\left(2011-2023\right)}{\#\;\text{survived}\;\text{from}\;\text{LA}\;\left(2011-2023\right)}/\frac{\#\;\text{died}\;\text{from}\;\text{LA}\;\left(2001-2010\right)}{\#\;\text{survived}\;\text{from}\;\text{LA}\;\left(2001-2010\right)}\right)$. We evaluated the impact of the Workgroup Epoch on death from all LAs, ester-linked LAs, amide-linked LAs, lidocaine, and long-acting LAs (bupivacaine, ropivacaine, and levobupivacaine).

ROR comparisons were made with Yates continuity-corrected χ^2^ test, and confidence intervals were calculated using Baptista–Pike method in GraphPad Prism (San Diego, CA, USA). Simple comparisons for age and weight were made with the Mann–Whitney *U*-test. Rate comparisons were made with Fisher’s exact test. Descriptive numerical data are presented as median and ranges, and categorical data are presented as counts and percentages. LA dose is presented as median and interquartile range.

### Exploratory analysis of case reports

Finally, we evaluated citations provided in the FAERS database linked to individual reports of death. Two reviewers (MRF and MG) screened citations for manuscripts or abstracts published from 2011 until 2023. After manuscript identification, papers were evaluated to assess if an LA contributed to death. Reports of LAs as contaminants in primary drug overdoses (e.g. cocaine) were excluded. Where available, details were extracted including study design, age, weight, sex, LA of interest, LA dose, route of administration, administrating provider, and whether lipid emulsion treatment was used. Drug doses were evaluated for exceeding package insert recommendations using 300 mg for lidocaine, 500 mg for lidocaine with epinephrine,^11^ 175 mg for bupivacaine, 225 mg for bupivacaine with epinephrine,^12^ 300 mg for ropivacaine,^13^ and 7 mg kg^−1^ for mepivacaine.^14^

## Results

### Food and Drug Administration Adverse Event Reporting System extraction

A total of 22 050 AEs related to isolated LAs from 1968 to 2023 were identified (Table 1) (Supplementary Fig. S1a) along with 1473 reports with ‘died’ as an outcome (Supplementary Fig. S1b). During the same period, there were 2 574 800 total reported deaths and 15 600 205 total reported AEs. The median age of patients across all reported LA events was 47 yr (range, 0–101 yr, *n*=14 961) compared with 50 yr (range, 0–101 yr, *n*=1023) among reports with an outcome of died (Mann–Whitney *U*-test, *P*=0.03). The median overall weight for LA reports was 69 kg (range, 0.45–220 kg, *n*=4236) compared with 62 kg (range, 0.45–159 kg, *n*=188) for reports with an outcome of died (Mann–Whitney *U*-test, *P*=0.0002). Overall AEs were reported in 6871 males and 10 945 females. Death was reported in 514 males and 680 females. Therefore, although AEs were reported more frequently in females, the likelihood of an outcome of died was higher in males (7.5% *vs* 6.3%**,** Fisher’s exact test *P*=0.0024).

**Table 1:** Reporting odds ratio of death from local anaesthetics relative to other entries in the US FDA Adverse Event Reporting System from 1968–2023 and 2019–2023. 95% CI, 95% confidence interval; FDA, Food and Drug Administration. ∗χ^2^ test. ^†^Local anaesthetics without a 2019–2023 row did not have any reports of death from 2019–2023.

		Death	Adverseevents	% Death	Reporting oddsratio of death (95% CI)	*P*-value∗
All reports	All years	2 574 800	15 600 205	16.50		
	2019–2023	888 813	6 087 930	14.60		
Amides	All years	1350	18 383	7.34	0.40 (0.37–0.42)	<0.001
	2019–2023	270	3083	8.76	0.56 (0.49–0.63)	<0.001
Lidocaine	All years	813	8210	9.90	0.55 (0.51–0.59)	<0.001
	2019–2023	212	1504	14.10	0.95 (0.83– 1.1)	0.605
Bupivacaine	All years	344	4719	7.29	0.39 (0.35–0.44)	<0.001
	2019–2023	36	1078	3.34	0.20 (0.14–0.28)	<0.001
Mepivacaine	All years	78	1881	4.15	0.21 (0.17–0.27)	<0.001
	2019–2023	7	143	4.90	0.30 (0.14–0.64)	0.002
Prilocaine^†^	All years	15	1381	1.09	0.05 (0.03–0.09)	<0.001
Ropivacaine	All years	76	1108	6.86	0.37 (0.29–0.47)	<0.001
	2019–2023	10	170	5.88	0.36 (0.19–0.69)	0.002
Articaine^†^	All years	7	651	1.08	0.05 (0.02–0.11)	<0.001
	2019–2023	4	162	2.47	0.14 (0.05–0.39)	<0.001
Etidocaine^†^	All years	9	306	2.94	0.15 (0.07–0.29)	<0.001
Levobupivacaine	All years	8	127	6.30	0.34 (0.16–0.69)	0.003
	2019–2023	1	26	3.85	0.23 (0.03– 1.7)	0.202
Esters	All years	41	1715	2.39	0.12 (0.09–0.16)	<0.001
	2019–2023	7	142	4.93	0.30 (0.14–0.64)	0.002
Benzocaine	All years	23	743	3.10	0.16 (0.10–0.24)	<0.001
	2019–2023	7	69	10.14	0.66 (0.30– 1.4)	0.380
Chloroprocaine^†^	All years	4	360	1.11	0.05 (0.02–0.15)	<0.001
Proparacaine^†^	All years	2	351	0.57	0.02 (0.00–0.11)	<0.001
Tetracaine^†^	All years	10	162	6.17	0.33 (0.17–0.63)	<0.001
Procaine^†^	All years	2	99	2.02	0.10 (0.02–0.42)	<0.001
Mixed	All years	82	1952	4.20	0.22 (0.17–0.27)	<0.001
	2019–2023	16	428	3.74	0.22 (0.13–0.37)	<0.001

### Adverse events with death as an outcome by drug class

**Fig 1:**
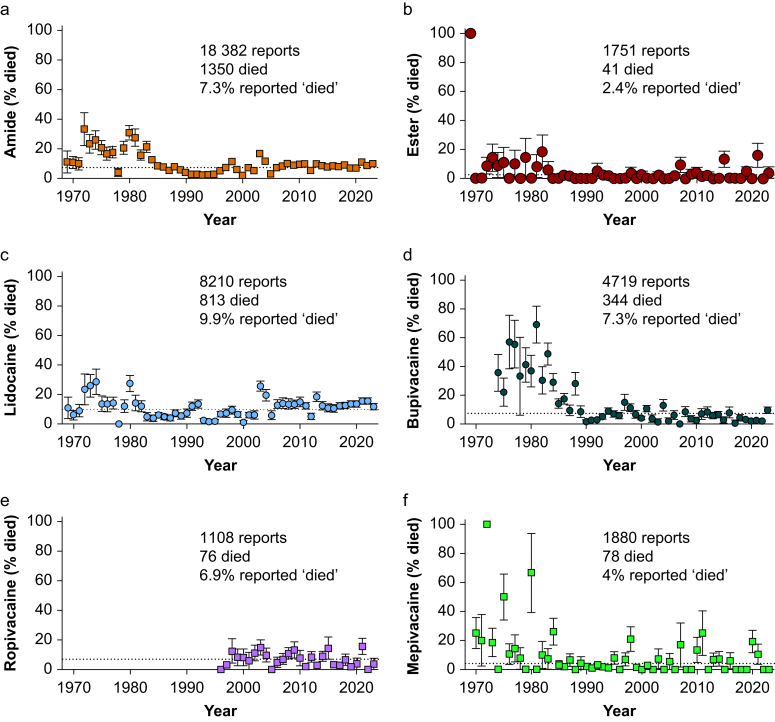
Percentage of serious adverse events reported with an outcome of ‘died’ (by year) for local anaesthetics in the US Food and Drug Administration Adverse Event Reporting System from 1968–2023. Confidence intervals calculated based on binomial distribution. (a) Percentage of amide-linked local anaesthetic cases with outcome of ‘died’. (b) Ester-linked local anaesthetic cases. (c) Lidocaine cases. (d) Bupivacaine cases. (e) Ropivacaine cases. (f) Mepivacaine cases.

AEs from amide-linked LAs were reported more frequently (Fig. 1a) than AEs from ester-linked LAs (Fig. 1b). Lidocaine had the highest rate of reporting of ‘died’ as an AE (9.9%, Fig. 1c), followed by bupivacaine (7.3%, Fig. 1d), ropivacaine (6.9%, Fig. 1e), levobupivacaine (6.3%, Supplementary Fig. S2a), mepivacaine (4.2%, Fig. 1f), etidocaine (2.9%, Supplementary Fig. S2b), prilocaine (1.1%, Supplementary Fig. S2c), and articaine (1.1%, Supplementary Fig. S2d). Among ester-linked LAs, tetracaine exhibited the highest rate of reporting of ‘died’ as an AE (6.2%, Supplementary Fig. S3a), followed by benzocaine (3.1%, Supplementary Fig. S3b), procaine (2.0%, Supplementary Fig. S3c), chloroprocaine (1.1%, Supplementary Fig. S3d), and proparacaine (0.6%, Supplementary Fig. S3e).

### Odds of death as a reported adverse event by drug class and epoch

**Fig 2:**
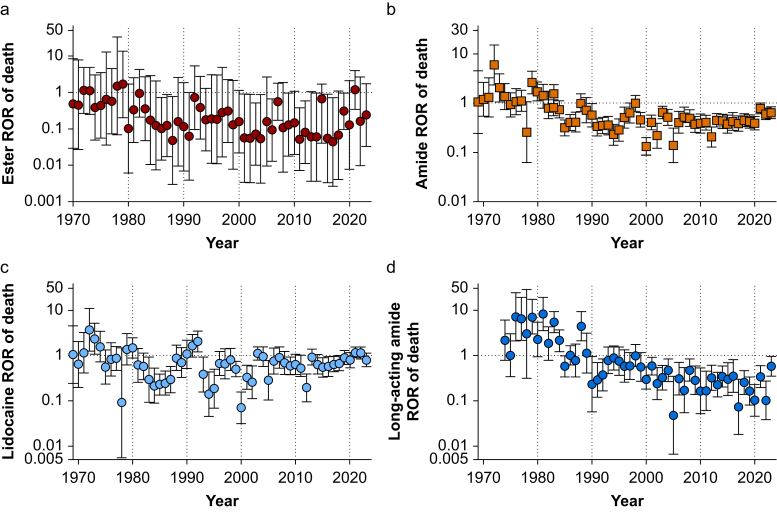
Reporting odds ratio of mortality by year relative to other entries in the Food and Drug Administration Adverse Event Reporting System. Reporting odds of death for (a) ester-linked local anaesthetics, (b) amide-linked local anaesthetics, (c) lidocaine, and (d) long-acting amide-linked local anaesthetics (bupivacaine, ropivacaine, levobupivacaine). ROR, reporting odds ratio.

From 1968 until present, ester-linked LAs were associated with a reduced ROR of death (ROR_death_) compared with other drugs in the database (ROR_death_=0.12, Table 1, Fig. 2a). Amide-linked LAs were also associated with a reduced ROR of death compared with non-LA drugs in the database (ROR_death_=0.4, Table 1, Fig. 2b). For the same time period, amide-linked LAs were associated with a higher ROR of death compared with ester-linked LAs (ROR_death_=3.2, 95% confidence interval [95% CI], 2.3–4.4, χ^2^=59, *P*<0.0001). For the most recent 5 yr of reporting (2019–2023), most LAs demonstrated lower ROR of death compared with other drugs in the database. The exceptions to this were lidocaine, benzocaine, and levobupivacaine (Table 1).

**Fig 3:**
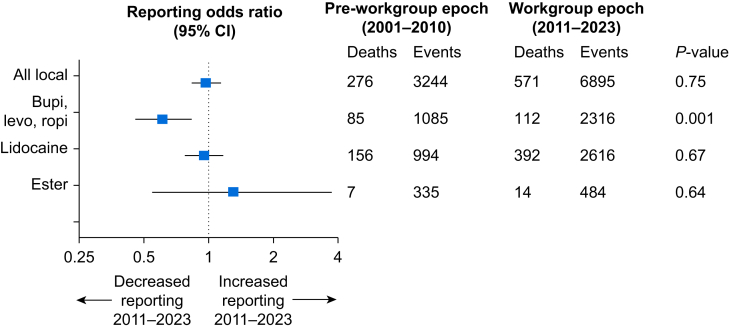
Reporting odds ratio of mortality comparing the Pre-Workgroup Epoch (2001–2010) with the Workgroup Epoch (2011–2023). Events calculated for all local anaesthetic reports, reports of long-acting local anaesthetics (bupi, bupivacaine; levo, levobupivacaine; ropi, ropivacaine), lidocaine and ester-linked local anaesthetics. Odds of mortality compared using the χ^2^ test. 95% CI, 95% confidence interval.

**Fig 4:**
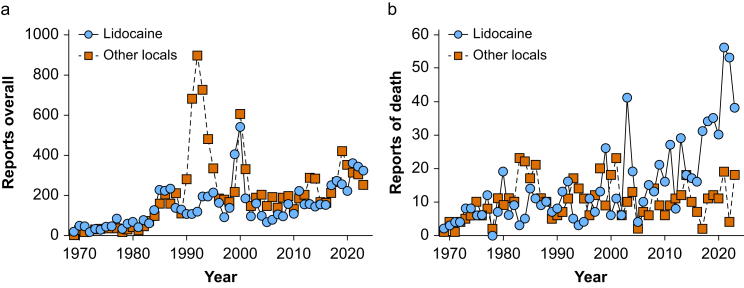
Local anaesthetic-associated events reported to the US Food and Drug Administration (FDA) Adverse Event Reporting System (a) Serious adverse events reported by year grouped into lidocaine and all other local anaesthetics (bupivacaine, ropivacaine, mepivacaine, chloroprocaine, procaine, etidocaine, prilocaine, articaine, benzocaine, tetracaine, proparacaine and levobupivacaine). (b) Serious adverse events with outcome of ‘died’ reported to the FDA Adverse Event Reporting System by year grouped by lidocaine and other local anaesthetics.

During the Workgroup Epoch, the ROR of death from amide-linked LAs or ester-linked LAs remained unchanged (Fig. 4). Similarly, lidocaine exhibited no change in ROR of death (Fig. 2c), but there was a reduction in ROR of death from long-acting LAs (Fig 2, Fig 3). Since the inception of the FAERS database (1968–2023), total reports of lidocaine correlated with total reports of other LAs. There is one region of discordance of increased reporting of prilocaine from 1990 to 1995 (Fig. 4a). Reports of death from lidocaine and death from other LAs were correlated until 2010. Starting in 2010, reports of deaths from lidocaine increased while those from other LAs remained flat (Fig. 4B). Over the past 3 yr, there was an average of 49 reports of death per year from lidocaine, but an average of 13 reports of deaths per year from all other LAs.

### Exploratory analysis of deaths

**Fig 5:**
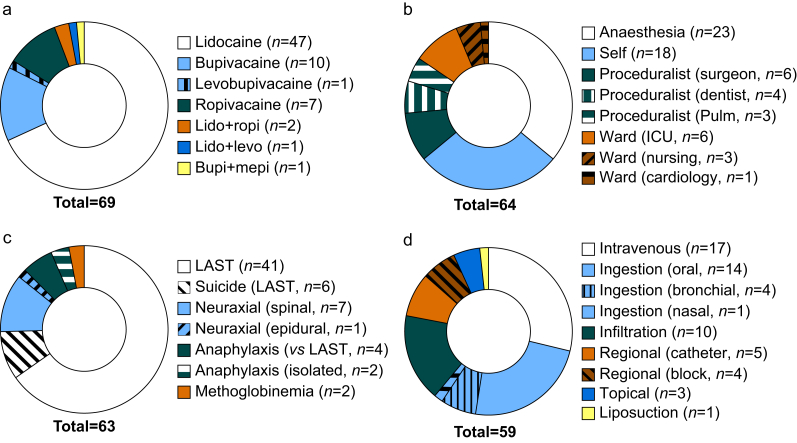
Extracted details of local anaesthetic-associated death from publications referenced in the Food and Drug Administration (FDA) Adverse Event Reporting System. Data include total number of cases and count (*n*) of individual causes. (a) Local anaesthetics associated with death. (b) Provider service administering local anaesthetic. (c) Inciting cause of harm by local anaesthetic. (d) Route of local anaesthetic administration. LAST, local anaesthetic systemic toxicity; Levo, levobupivacaine; Lido, lidocaine; Mepi, mepivacaine; Pulm, pulmonologist; Ropi, ropivacaine.

After screening, we identified 57 papers with 207 deaths from LAs published since 2011. The papers included two population health studies with unclear causation which were excluded from analysis.^15^^,^^16^ It also included 41 cases reported over 12 yr by the American Poison Centers National Poison Data System,^17^, ^18^, ^19^, ^20^, ^21^, ^22^, ^23^, ^24^, ^25^, ^26^, ^27^, ^28^ and previously analysed elsewhere.^29^ We analysed 43 case reports, case series, and retrospective reviews reporting 69 deaths (Supplementary Table S1).^30^, ^31^, ^32^, ^33^, ^34^, ^35^, ^36^, ^37^, ^38^, ^39^, ^40^, ^41^, ^42^, ^43^, ^44^, ^45^, ^46^, ^47^, ^48^, ^49^, ^50^, ^51^, ^52^, ^53^, ^54^, ^55^, ^56^, ^57^, ^58^, ^59^, ^60^, ^61^, ^62^, ^63^, ^64^, ^65^, ^66^, ^67^, ^68^, ^69^, ^70^, ^71^, ^72^ Of those 69 cases, lidocaine was the most frequent cause of death (74% of cases, Fig. 5a). Anaesthesia was the most frequent provider (36% of cases) but also with delivery by proceduralists and on wards (Fig. 5b). LAST was the most common complication (75% of cases, Fig. 5c). Delivery was most frequently i.v. (29%) or by ingestion (32%, Fig. 5d). Of cases with reported doses, 61% of lidocaine administrations exceeded package insert recommendations with a median dose of 640 mg (interquartile range [IQR], 85–3456 mg, *n*=30), 44% of ropivacaine administrations with a median dose of 280 mg (IQR, 113–400 mg, *n*=9), and 0% of bupivacaine administrations with a median dose of 17.5 mg (IQR, 11.4–24, *n*=10). High-dose oral lidocaine as a suicide attempt represented the most frequently reported cause of death (*n*=14 [20%], often multiple grams), followed by high-dose lidocaine i.v. (*n*=13 [19%], often multiple grams), endobronchial lidocaine in sick patients (*n*=4 [6%]), ropivacaine or bupivacaine nerve catheters (*n*=5, [7%]), and bupivacaine spinals (*n*=7, [10%]). Sixteen cases (23%) occurred in out-of-hospital settings, and authors noted lipid emulsion or fat emulsion administration in only eight (12%) cases. Deaths that occurred in the setting of regular dosing of local anaesthesia included high spinals, endobronchial delivery of lidocaine (with challenges estimating total doses and rapid uptake from mucus membranes), anaphylaxis, methaemoglobinemia, and sodium channel mutations.

## Discussion

Our evaluation of the FAERS database identified that LAs have a reasonably safe profile, with death reported less frequently as an AE than for other drugs in the database. The reporting rates of death of 1–10% comport with previous LAST case reviews where two to five of every 50 LAST cases resulted in a death.^2^^,^^73^, ^74^, ^75^ Our study identified that reporting of death from long-acting LAs (bupivacaine, ropivacaine, and levobupivacaine) decreased during the Workgroup Epoch,^5^^,^^6^ but death persisted from nerve catheters and high spinals, risks identified elsewhere.^75^, ^76^, ^77^, ^78^ Unexpectedly, lidocaine was reported most frequently as a source of death, with high-dose oral and i.v. delivery contributing. The perceived safety of lidocaine might lead providers to administer doses in excess of the package insert, contributing to catastrophic events when toxicity occurs. Case reports also detailed out-of-hospital suicidal/homicidal events involving large doses of viscous lidocaine, easily purchased in multiple-gram quantities over the internet. These findings identify opportunities for heightened awareness amongst providers and openings for professional societies or regulatory agencies to provide recommendations to reduce LA-associated deaths. This includes guidance for oropharyngeal topicalisation, restrictions on access to large doses of outpatient lidocaine, advice on dose limits for peripheral nerve catheters,^75^^,^^77^ interventions to minimise high spinal blocks, and the advocacy for safer anaesthetics such as articaine over lidocaine.^79^

### Relative mortality

Since 2010, the odds of reporting death from long-acting LAs have decreased relative to the previous decade. An outcome of ‘died’ was only reported in 3.4% of bupivacaine AEs over the past 5 yr compared with an overall rate of 7.3% (1968–2023), reflecting the reduction in reporting of death from bupivacaine. Our review of cases identified only four nerve blocks that contributed to death since 2010. All used LA doses exceeding the package insert recommendations or LA mixtures. This included 800 mg of lidocaine,^66^ 112.5 mg of ropivacaine mixed with 300 mg of lidocaine,^46^ 25 mg of bupivacaine (0.55 mg kg^−1^) mixed with 225 mg of mepivacaine (5 mg kg^−1^),^67^ and 100 mg of lidocaine mixed with 37.5 mg of bupivacaine.^62^ Surprisingly, high neuraxial block was the primary cause of death from bupivacaine in the cases we evaluated. Other delivery modes contributing to death included ropivacaine catheters, oropharyngeal and endobronchial lidocaine topicalisation, i.v. lidocaine (or ropivacaine), and completed suicides utilising viscous lidocaine. The cited literature in the FAERS database failed to capture case reports of death from benzonatate,^80^ articaine,^81^ tetracaine,^82^ or i.v. lidocaine,^83^ along with retrospective reviews describing deaths in dentistry (primarily from lidocaine),^84^ French pharmacovigilance studies identifying deaths from lidocaine,^85^^,^^86^ and multiple publications of death from high spinal blocks.^87^, ^88^, ^89^

Several changes, both systemic and behavioural, occurred around 2010 to reduce the risk of death from single-shot injection of long-acting LAs. Firstly, multiple regional anaesthesia societies introduced practice advisories on the prevention and treatment of LAST.^5^^,^^6^ These advisories included a recommendation to use lipid emulsion for LAST. Before the advent of lipid emulsion therapy, cases of bupivacaine toxicity often required prolonged resuscitation and extreme measures such as venoarterial extracorporeal membrane oxygenation or cardiopulmonary bypass. Since the introduction of lipid emulsion, there have been few cases of bupivacaine toxicity requiring cardiopulmonary bypass.^90^ Secondly, 2010 coincided with the widespread adoption of ultrasound for regional anaesthesia, and the data suggest that these developments in the early 2010s reduced reports of death from long-acting LAs.

### Reporting of death associated with lidocaine

Curiously, reports of death associated with lidocaine have not followed the same trajectory as other LAs. Over the past 5 yr, lidocaine exhibited the highest ROR of death for any LA in the database. Previous authors identified the risk of lidocaine in liposuction,^91^ leading to recommendations about upper dose limits. Previous studies also identified the risk of lidocaine as a source of death in dental procedures,^84^ contributing to the clinical introduction of articaine to dental practice in 2000. Beyond this, the American Academy of Pediatric Dentists introduced upper limits for LA doses in dental procedures, including for lidocaine.^92^ Many of these interventions should have made lidocaine use safer, raising the question of why reporting of death from lidocaine persists.

Multiple changes in practice spurred a transition to increased use of lidocaine in the past 20 yr. In the wake of the opioid crisis, practitioners embraced opioid-free anaesthesia, which incorporates multiple lidocaine-based interventions, including patches, i.v. infusions, and delivery via peripheral nerve catheters. Because of the perceived safety of lidocaine, practitioners do not consistently monitor for toxic events, and both surgeons and proceduralists frequently use lidocaine with other LAs, increasing the risk of toxicity. Even in the absence of other LAs, retrospective safety studies identified mortality at one in 4000 patients receiving postoperative lidocaine infusions.^52^ The danger of lidocaine infusions is unsurprising given that lidocaine used for ventricular arrhythmias after myocardial infarction contributes to mortality.^93^ A multidisciplinary group of experts from the UK and Canada recently provided recommendations for perioperative lidocaine infusions,^94^ but the impact of these recommendations remains uncertain.

Topical lidocaine, including patches and creams, represents another source of death. High-concentration lidocaine patches were introduced in 1999 with the assertion of a high margin of safety owing to low transdermal uptake. Their use has proliferated to practices not recommended by the manufacturer, including a case report of 10 patches daily for 23 months.^95^ The FAERS data also contradict the assertions of safety with 77 reports of death from Lidoderm® (Teikoku Pharma, Kagawa, Japan) in the FAERS, along with 23 reports of death from viscous lidocaine, 17 reports from lidocaine patches (generic), and five reports from Solarcaine cool aloe® (Wellspring Pharmaceutical Corporation, Sarasota, FL, USA). A recent analysis from the US National Poison Data System also identified two paediatric cases of death from topical lidocaine.^43^ Compared with the 1980s, an era when bupivacaine was feared for its cardiotoxicity, the past 10 yr are dominated by death from lidocaine.^31^^,^^32^^,^^42^^,^^44^^,^^48^^,^^56^^,^^66^^,^^96^

### Improving the safety of local anaesthetics

Improving the safety of LAs will likely require changes to clinical practice for using lidocaine. This includes recognition that i.v. infusion of lidocaine^52^ and truncal infusion of lidocaine^97^ carry similar risks of accumulation as bupivacaine or ropivacaine.^75^ The most recent Cochrane review questioned the benefit of perioperative lidocaine infusions.^98^ With reports of death in association with lidocaine infusions,^52^^,^^96^ perioperative physicians should reevaluate the practice. The indiscriminate use of i.v. lidocaine as part of an induction cocktail for general anaesthesia also requires reconsideration given reports of AEs in at-risk patients.^99^ Many of the AEs associated with lidocaine occur in prehospital environments, emergency departments, and non-anaesthetising procedural locations. Recommendations are needed for the use of large doses of oropharyngeal or endobronchial lidocaine, along with a review of access to large doses of outpatient lidocaine. The field of medicine, as a whole, could benefit from alternatives to lidocaine, such as ester-linked LAs that do not accumulate and are metabolised by plasma esterases.

Dosing recommendations are needed for ropivacaine and bupivacaine catheters, both by intermittent bolus^77^ and by continuous infusion.^75^^,^^76^ Providers would benefit from strategies to prevent and treat high neuraxial anaesthesia in obstetric^78^ and other populations. Recommendations and guidelines should encourage the use of novel compounds that have a less toxic profile, including Nav 1.8 blockers and capsaicinoids. Finally, education of other procedural specialties might help reduce AEs associated with LAs.

### Limitations

This work has several limitations, primarily driven by limitations of the FAERS database, including incomplete reporting (on age, sex, and comorbidities), potential underreporting of events, possible double-reporting of events, lack of verification of events, and lack of causality. It represents a single source of pharmacovigilance data from a single country about AEs that requires verification and corroboration from other sources.^29^ Many of these alternative sources support the conclusion about the frequency of lidocaine-associated events, including case reports,^31^^,^^32^^,^^42^^,^^44^^,^^48^^,^^56^^,^^66^^,^^96^ case reviews,^2^^,^^74^ and pharmacovigilance studies.^29^^,^^86^^,^^100^

### Conclusions

Compared with other drugs, local anaesthetics are reported infrequently to the US Food and Drug Administration Adverse Event Reporting System (FAERS) registry as a cause of death. Reports of death as an AE from bupivacaine has decreased from its peak in the 1980s; the introduction of practice advisories in 2010^5^^,^^6^ was associated with a further decrease in reporting of death from long-acting local anaesthetics. However, reporting of death from lidocaine to FAERS has remained unchanged over the past 20 yr. The persistence of lidocaine-associated mortality reinforces the danger of lidocaine identified previously^29^^,^^86^^,^^100^ and merits further study. Interventions to improve safety include overall dosing limit algorithms (taking weight, comorbidities and repeat dosing into account),^75^^,^^77^ specific recommendations for use in various clinical situations,^94^ reinforcing the use of lipid emulsion to treat toxicity, and rigorous education of providers to reduce mortality associated with local anaesthetic use.

## Authors’ contributions

Conceived the study: MRF

Acquired the data, analysed the data, and drafted the manuscript: MRF, MG

Contributed to interpretation of the data, editing, and revision, and agree to the final version and to be accountable for all aspects of the work: all authors

## Funding

University of Illinois Health Department of Anesthesiology, Chicago, IL, USA (to MRF and HCN).

## Declarations of interest

GW is an officer and shareholder of ResQ Pharma, Inc (Chicago, IL, USA). The other authors declare that they have no conflicts of interest.
