# Supplementary material for: Contemporary local anaesthetic-associated adverse events and mortality: a pharmacovigilance analysis of a US reporting system
Source: Br J Anaesth. 2025 Aug 27;135(4):1015–25. doi: 10.1016/j.bja.2025.06.044 (PMC12674073; doi:10.1016/j.bja.2025.06.044)
Supplement: Multimedia component 1 [file mmc1.docx]

**Supplemental Material: Contemporary local anaesthetic associated mortality**

Michael R. Fettiplace MD PhD, Guy Weinberg MD, Christopher Chiang MD, Heather C. Nixon MD, Marina Gitman MD

**Supplemental methods**

**Search terms**

We used the following dropdown menu search terms:*:* “Bupivacaine (G)”,“Bupivacaine Hydrochloride (G)”, “Bupivacaine Hydrochloride\Epinephrine(G)”, “Bupivacaine\Epinephrine (G)”, “Bupivacaine Hydrochloride\Epinephrine Hydrochloride(G); “Ropivacaine (G), “Ropivacaine Hydrochloride (G)”, “Epinephrine\Ropivacaine”, “Epinephrine\Ropivacaine Hydrochloride (G)”, “Ropivacaine Hydrochloride Anhydrous (G)”; “Lidocaine (G)”, “Lidocaine Hydrochloride (G)”, “Lidocaine Carbonate (G)”, “Epinephrine\Lidocaine”, “Epinephrine\Lidocaine Hydrochloride (G)”; “Mepivacaine Hydrochloride (G)”, “Epinephrine\Mepivacaine (G)”, “Epinephrine\Mepivacaine Hydrochloride (G)”, “Mepivacaine Hydrochloride\Norepinephrine Bitartrate (G)”; “Chloroprocaine (G)”, “Chloroprocaine Hydrochloride (G)”, “Procaine (G)”, “Procaine Hydrochloride (G)”, “Epinephrine/Procaine (G)”, “Epinephrine/Procaine Hydrochloride (G)”, “Epinephrine bitartrate/etidocaine hydrochloride (G)”, “Etidocaine (G)”, “Etidocaine hydrochloride (G)”, “Prilocaine (G)”, “Prilocaine hydrochloride (G)”, “Epinephrine bitartrate/prilocaine hydrochloride (G)”, “Epinephrine/prilocaine (G)”, “Articaine (G)”, “Articaine hydrochloride (G)”, “Articaine hydrochloride/Epinephrine bitartrate (G)”, “Articaine hydrochloride/epinephrine hydrochloride (G)”, “Articaine/epinephrine (G)”, “Benzocaine (G)”, “Benzocaine hydrochloride (G)”, “Tetracaine (G)”, “Tetracaine hydrochloride (G)”, “Proparacaine (G)”, ”Proparacaine hydrochloride (G)”, “Levobupivacaine (G)”, “Levobupivacaine hydrochloride (G)”.

**Supplemental Figure Legends**

**Supplemental figure 1:** Extraction flow chart for data retrieved from FDA adverse event reporting system

**Supplemental Figure 2:** Percent of serious adverse events reported with an outcome of “died” (by year) for amide-linked local anaesthetics in the FDA adverse event reporting system from 1968 – 2023; confidence intervals calculated based on binomial distribution. **A.** Percent of levobupivacaine (levobupi) cases with outcome of died **B.** Etidocaine cases **C.** Prilocaine cases **D.** Articaine cases

**Supplemental Figure 3:** Percent of serious adverse events reported with an outcome of “died” (by year) for ester-linked local anaesthetics in the FDA adverse event reporting system from 1968 – 2023; confidence intervals calculated based on binomial distribution. **A.** Percent of Tetracaine cases with an outcome of “died” **B.** Benzocaine cases **C.** Procaine cases **D**. Chloroprocaine cases **E.** Proparacaine cases

**References**

1. Alexandre J, Humbert X, Sassier M, et al. Cardiac arrests associated with low plasma and tissue levels of local anaesthetics. *Therapies* Elsevier Masson s.r.l.; 2015; 78: S81–4

2. Maedeh Asna Ashari Amiri, Fatemeh Mohammadi, Seyed Amir Tabibzadeh Dezfooli, Mahya Naderkhani. Fatal Systemic Toxicity with Topical Lidocaine Spray. *J Pharm Negat Results* Siree Journals; 2022; 611–3

3. Attafi IM, Oraiby ME, Albeishy MY, Fageeh MM. Postmortem Redistribution of Lidocaine after Illegal Use. *The Saudi Journal of Forensic Medicine and Sciences* Medknow; 2023; 3: 21–4

4. Barbera N, Busardò FP, Indorato F, Romano G. The pathogenetic role of adulterants in 5 cases of drug addicts with a fatal outcome. *Forensic Sci Int* Elsevier Ireland Ltd; 2013; 227: 74–6

5. Bazerbachi F, Rank K, Chan A. Intravenous lipid rescue and ropivacaine systemic toxicity. J Anesth. 2014. p. 139

6. Busardò FP, Tritapepe L, Montana A, Indorato F, Zaami S, Romano G. A fatal accidental subarachnoid injection of lidocaine and levobupivacaine during a lumbar paravertebral block. *Forensic Sci Int* Elsevier Ireland Ltd; 2015; 256: 17–20

7. Calenda E, Baste JM, Hajjej R, Danielou E, Peillon C. Toxic plasma concentration of ropivacaine after a paravertebral block in a patient suffering from severe hypoalbuminemia. *J Clin Anesth* Elsevier Inc.; 2014; 26: 149–51

8. Chan TYK. Fatal anaphylactic reactions to lignocaine. Forensic Sci Int. Elsevier Ireland Ltd; 2016. p. 449–52

9. Chassard D. Mortalité maternelle liée à l’anesthésie-réanimation. Résultats de l’ENCMM, France 2010–2012. *Gynecologie Obstetrique Fertilite et Senologie* Elsevier Masson SAS; 2017; 45: S54–7

10. Cole JB, Olives TD, Ulici A, et al. Extracorporeal Membrane Oxygenation for Poisonings Reported to U.S. Poison Centers from 2000 to 2018: An Analysis of the National Poison Data System*. *Crit Care Med* 2020; 48: 1111–9

11. Coquerel A, Cesbron A, Fedrizzi S, et al. Is there an underestimated toxicity of local anaesthetics (LA) as antalgics during or after surgery? Two cases of sudden death with non-toxic level of LA in blood and tissues. *Basic Clin Pharmacol Toxicol* [Internet] 2014; 115: 54–91 Available from: https://onlinelibrary.wiley.com/doi/10.1111/bcpt.12259_2

12. Drapkin Z, Caravati EM, Plumb J, Szadkowski M. 174. Intentional oral viscous liodcaine overdose resulting in seizure and death in an adolescent (Abstract). *Clin Toxicol* 2015; 53: 719–20

13. Eisenberg J, Tedford NJ, Weaver N, Becker S, Moss MJ. Adverse Outcomes in Topical Lidocaine Exposure: A Pediatric Case Series From the United States National Poison Data System. *Clin Pediatr (Phila)* SAGE Publications Inc.; 2023; 62: 1390–7

14. Fagenholz PJ, Bowler GMR, Carnochan FM, Walker WS. Systemic local anaesthetic toxicity from continuous thoracic paravertebral block. *Br J Anaesth* Oxford University Press; 2012; 109: 260–2

15. Gaballah MH, Tobaigi MS, Hakami A, et al. Autopsy findings and toxicological results of two cases of sudden death due to intoxication by lidocaine. *Forensic Science International: Reports* Elsevier B.V.; 2022; 6

16. Gay HC, Amaral AP. Acquired Methemoglobinemia Associated with Topical Lidocaine Administration: A Case Report. *Drug Saf Case Rep* Springer Nature; 2018; 5

17. Gicquel-Schlemmer B, Beller JP, Mchalwat A, Gicquel P. Fatal Takotsubo cardiomyopathy due to epinephrine in shoulder arthroscopy. *Orthopaedics and Traumatology: Surgery and Research* Elsevier Masson SAS; 2015; 101: 981–2

18. Kantarci MN, Kandemir E, Balkay M, et al. Descriptive features of depressive disorder patients with who have committed suicide by drug overdose. *Klinik Psikofarmakoloji Bulteni* Cukurova Univ Tip Fakultesi Psikiyatri Anabilim Dali; 2013; 23: 155–61

19. Kaya K, Mungan U. Severe methemoglobinemia following intravenous lidocaine administration during coronary artery bypass surgery: The use of methylene blue and extracorporeal membrane oxygenator. *Turkish Journal of Thoracic and Cardiovascular Surgery* Baycinar Medical Publishing; 2021; 29: 546–8

20. Kirschner R, Smith K. 61. Fatal Dysrhythmia Associated with Subcutaneous Ropivacaine Infusion in a Child. *Clin Toxicol* 2019; 57: 903–903

21. Kogulshankar M, Mayorathan U, Pranavan S, Munasinghe BM. Fatality in the perioperative period: A case report on refractory hypoxemia in an infant following open inguinal herniotomy. *Int J Surg Case Rep* Elsevier Ltd; 2023; 106

22. Kradel BK, Hinson SB, Smith CJ. Two obese patients with presumptive diagnosis of anaphylactoid syndrome of pregnancy presenting at a community hospital. *American Journal of Case Reports* Medical Science International; 2016; 17: 444–7

23. Miller M, Jayaram J, Allen BFS, Freundlich RE, Wanderer JP, McEvoy MD. Safety of postoperative lidocaine infusions on general care wards without continuous cardiac monitoring in an established enhanced recovery program. *Reg Anesth Pain Med* BMJ Publishing Group; 2022;

24. Mittal S, Mohan A, Madan K. Ventricular Tachycardia and Cardiovascular Collapse following Flexible Bronchoscopy: Lidocaine Cardiotoxicity. *J Bronchology Interv Pulmonol* Lippincott Williams and Wilkins; 2018; 25: e24–6

25. Moellentin DL, Stewart D, Barbour J. Case study of fatal stroke following intranasal lidocaine. *Hosp Pharm* Thomas Land Publishers Inc.; 2016; 51: 662–4

26. Morcos A, Brault S, Dumestre-Toulet V. Décès d’un nourrisson après injection de lidocaïne. *Toxicologie Analytique et Clinique* [Internet] 2023; 35: S21–S21 Available from: https://doi.org/10.1016/j.toxac.2023.03.024Get

27. Mrad S, Tawil C El, Sukaiti WA, Chebl RB, Dagher GA, Kazzi Z. Cardiac Arrest Following Liposuction: A Case Report of Lidocaine Toxicity. *Oman Med J* 2019; 34: 341–4

28. Nefcy A, Malone E, King A, Aaron C. Fatal Intoxication of Twin Girls After Ingestion of Topical 2% Viscous Lidocaine (Abstract). *Journal of Medical Toxicology* NLM (Medline); 2017; 13: 30–30

29. Pélissier-Alicot AL, Deveaux M, Sastre C, et al. Planned complex suicide involving combined drug intoxication and femoral catheterization. *J Forensic Sci* John Wiley and Sons Inc; 2021; 66: 2527–31

30. Pigolkin YI, Shigeev S V., Denisova A V., Natarova K V., Krupin KN. Forensic medical assessment of lidocaine and bupivacaine systemic toxicity. *Sudebno-Meditsinskaya Ekspertiza* Media Sphera Publishing Group; 2023; 66: 62–6

31. Pitkänen MT, Aromaa U, Cozanitis DA, Förster JG. Serious complications associated with spinal and epidural anaesthesia in Finland from 2000 to 2009. *Acta Anaesthesiol Scand* 2013; 57: 553–64

32. Rahimi M, Elmi M, Hassanian-Moghaddam H, et al. Acute Lidocaine Toxicity; a Case Series [Internet]. Emergency. 2018 Available from: www.jemerg.com

33. Spitzer D, Wenger KJ, Neef V, et al. Local anesthetic-induced central nervous system toxicity during interscalene brachial plexus block: A case series study of three patients. *J Clin Med* MDPI; 2021; 10: 1–13

34. Sriramatr D, Chongarunngamsang W, Plansangkate P, et al. Cardiac arrest and complications after spinal anesthesia: The perioperative and anesthetic adverse event in Thailand (PAAd Thai) incident report study. *Journal of the Medical Association of Thailand* Medical Association of Thailand; 2021; 104: 663–71

35. Sud A, Szawarski P. Classic cases revisited – Death of a nurse and the anatomy of error. *J Intensive Care Soc* SAGE Publications Inc.; 2018; 19: 155–60

36. Szadkowski M, Drapkin Z, Hewes H, Caravati EM, Plumb J. A Teenager with Seizures and Cardiac Arrest after Drug Overdose: Are We Numb to the Danger? *Pediatr Emerg Care* Lippincott Williams and Wilkins; 2017; 33: 657–9

37. Téllez-Peña DM, Martínez-González ME, Andrade-Caballero A. Systemic intoxication by local anesthetics. *Revista Mexicana de Anestesiologia* Colegio Mexicano de Anestesiologia A.C.; 2022; 45: 207–11

38. Vadi MG, Patel N, Stiegler MP. Case Scenario: Local Anesthetic Systemic Toxicity after Combined Psoas Compartment-Sciatic Nerve Block: Analysis of Decision Factors and Diagnostic Delay. *Anesthesiology* 2014; 120: 987–96

39. van Zyl SF, Burke JL. Increasing the labour epidural rate in a state hospital in South Africa: Challenges and opportunities. *Southern African Journal of Anaesthesia and Analgesia* Medpharm Publications; 2017; 23: 23–8

40. Weeke LC, Schalkwijk S, Toet MC, Van Rooij LGM, De Vries LS, Van Den Broek MPH. Lidocaine-Associated Cardiac Events in Newborns with Seizures: Incidence, Symptoms and Contributing Factors. *Neonatology* S. Karger AG; 2015; 108: 130–6

41. Winkler M, Alt A, Dietzsch S, Kunz SN. Two fatal and four surviving cases after accidental infusion of ropivacaine. *Forensic Toxicol* Springer Japan; 2021; 39: 506–12

42. Winograd E, Bisoski L, Aaron C, Dimovska M. 132. Death Following Lidocaine Inhalation for Treatment of Gastroesophageal Reflux Disease (Abstract). *Journal of Medical Toxicology* NLM (Medline); 2018; 14: 49–49

43. Xiong Q, Cao L, Hu J, Marian AJ, Hong K. A rare loss-of-function SCN5A variant is associated with lidocaine-induced ventricular fibrillation. *Pharmacogenomics Journal* Nature Publishing Group; 2014; 14: 372–5
