# Supplementary material for: Contemporary local anaesthetic-associated adverse events and mortality: a pharmacovigilance analysis of a US reporting system
Source: Br J Anaesth. 2025 Aug 27;135(4):1015–25. doi: 10.1016/j.bja.2025.06.044 (PMC12674073; doi:10.1016/j.bja.2025.06.044)
Supplement: Multimedia component 2 [file mmc2.docx]

**Supplemental Table 1:** Extracted citations of local anaesthetic associated mortality from FDA-Adverse Event System

| **Citation** | **Local (#)** | **Age / Gender** | **Out-of-hospital** | **Route** | **Weight** | **Dose (mg)** | **ILE** | **Study design** | **Administering Service** | **Inciting Cause** |
| --- | --- | --- | --- | --- | --- | --- | --- | --- | --- | --- |
| Alexandre 2023^1^ | Lidocaine | 69 / F |  | IV | 84 | 479 |  | CR | Anesthesia | LAST |
| Amiri 2022^2^ | Lidocaine | / F | x | Ingestion (PO) | |  | Yes | CR | Self | LAST |
| Attafi 2021^3^ | Lidocaine | 30 / M | x | Ingestion (PO) | | 5000 |  | CS | Self | LAST |
|  | Lidocaine | 25 / F | x | LIA |  |  |  | CS | Surgeon | LAST |
| Barbera 2013^4^ | Lidocaine | 26 / M | x | IV |  |  |  | CS | Self | LAST |
| Bazerbachi 2014^5^ | Ropivacaine | 57 / M |  | Catheter |  | 1540 | Yes | CS | Anesthesia | LAST |
| Busardo 2015^6^ | Lidocaine, Levo-bupivacaine | 60 / F |  | Spinal |  | 10 , 10 |  | CR | Anesthesia | High spinal |
| Calenda 2014^7^ | Ropivacaine | 72 / M |  | Catheter | 70 | 280 |  | CR | Surgeon | LAST |
| Chan 2016^8^ | Lidocaine | 19 / F |  | Ingestion (EB) | | <300 |  | CS | Pulm | LAST |
|  | Lidocaine | 67 / M |  | Ingestion (EB) | | 80 |  | CS | Pulm | LAST vs anaphylaxis |
|  | Lidocaine | 20 / M |  | LIA |  | 16 |  | CS | Dentist | LAST vs anaphylaxis |
|  | Lidocaine | 43 / F |  | LIA |  | 50 |  | CS | Unknown | Anaphylaxis |
|  | Lidocaine | 44 / F |  | LIA |  | 1000 |  | CS | Surgeon | LAST vs anaphylaxis |
|  | Lidocaine | 4 / F |  | LIA |  | 18 |  | CS | Dentist | LAST vs anaphylaxis |
|  | Lidocaine | 50 / F |  | Neuraxial |  | 80 |  | CS | Unknown | Anaphylaxis |
| Chassard 2017^9^ | Ropivacaine | / F |  | Epidural / LIA | | 150 , unknown |  | RR | Anesthesia / Surgeon | LAST |
|  | Bupivacaine | 31 / F |  | Spinal |  | 10 |  | RR | Anesthesia/ pain | High spinal |
| Cole 2020^10, 11^ | Lidocaine | / |  |  |  |  |  | RR | |  |
|  | Lidocaine | / |  |  |  |  |  | RR | |  |
| Coquerel 2014^11^ | Levo-bupivacaine | 58 / F |  | Catheter |  |  |  | CS | Anesthesia |  |
|  | Lidocaine | 69 / F |  | IV |  |  |  | CS | Anesthesia |  |
| Drapkin 2015^12^ | Lidocaine | 15 / F |  | Ingestion (PO) | 41 | 2000 |  | CR | Self | LAST |
| Eisenberg 2023^13^ | Lidocaine | 18 / |  | Topical |  |  |  | RR | Self | LAST |
|  | Lidocaine | 18 / |  | Topical |  |  | Yes | RR | Self | LAST |
| Fagenholz 2012^14^ | Bupivacaine | 67 / M |  | Catheter | 39 | 1000+ |  | CR | Anesthesia | LAST |
| Gaballah 2022^15^ | Lidocaine | 32 / M | x | Ingestion (PO) | | 5000 |  | CS | Self | LAST |
|  | Lidocaine | 22 / F | x | Topical / LIA |  |  |  | CS | Surgeon | LAST |
| Gay 2018^16^ | Lidocaine | 55 / M |  | Ingestion (EB) | | 4% gel |  | Case study | Anesthesia | Methemo-globinemia |
| Gicquel-Schlemmer 2015^17^ | Ropivacaine, lidocaine | 48 / F |  | Block |  | 112.5 , 300 |  | CR | Anesthesia | Unclear |
| Kantarci 2013^18^ | Lidocaine | / |  | Ingestion (PO) | |  |  | RR | Self | Suicide |
|  | Lidocaine | / |  | Ingestion (PO) | |  |  | RR | Self | Suicide |
| Kaya 2021^19^ | Lidocaine | 63 / M |  | IV | 80 | 80 |  | CR | Anesthesia | Methemo-globinemia |
| Kirschner 2019^20^ | Ropivacaine | 4 / M |  | Catheter | 15.3 | 580 | Yes | CR | Anesthesia | LAST |
| Kogulshankar 2023^21^ | Bupivacaine | 0.083 / M |  | LIA | 2.9 | 5 |  | CR | Anesthesia | LAST |
| Kradel 2016^22^ | Bupivacaine | 29 / F |  | Spinal | 140 | 11.25 |  | CS | Anesthesia | High Spinal |
| Miller 2022^23^ | Lidocaine | / |  | IV |  | - |  | RR | Anesthesia | LAST (Lido 5.9mcrog/mL) |
| Mittal 2018^24^ | Lidocaine | 65 / M |  | Ingestion (EB) | 55 | 330 |  | CR | Pulm | LAST |
| Moellentin 2016^25^ | Lidocaine | 39 / M | x | Ingestion (nasal) | | 4800 |  | CR | Self | LAST |
| Morcos 2023^26^ | Lidocaine | 0.083 / M | x | LIA |  |  |  | CR | Surgeon | LAST |
| Mrad 2019^27^ | Lidocaine | 33 / F |  | Liposuction | 70 | 5000 |  | CR | Surgeon | LAST |
| Nefcy 2017^28^ | Lidocaine | 1.1 / | x | Ingestion (PO) | |  |  | CR | Self | LAST |
|  | Lidocaine | 1.1 / | x | Ingestion (PO) | |  |  | CR | Self | LAST |
| Pelissier-Alicot 2021^29^ | Lidocaine | 42 / M | x | IV |  |  |  | CR | Self | Suicide, LAST (lidocaine 7.3 mg/L) |
| Pigolkin 2023^30^ | Lidocaine | 29 / F |  | Ingestion (PO) | | 300 |  | CS | Dentist | LAST |
|  | Lidocaine | 25 / M |  | LIA |  |  |  | CS | Dentist | LAST |
|  | Bupivacaine | 37 / F |  | Spinal |  | 20 |  | CS | Anesthesia | High Spinal |
| Pitkanen 2013^31^ | Bupivacaine | 67 / M |  | Epidural |  | 37 |  | RR | Anesthesia | High Epidural |
|  | Ropivacaine | 20 / F |  | spinal |  | 18 |  | RR | Anesthesia | High spinal |
| Rahimi 2018^32^ | Lidocaine | 20 / M | x | Ingestion (PO) | | 250 |  | CS | Self | Suicide |
|  | Lidocaine | 21 / M | x | Ingestion (PO) | | 250 |  | CS | Self | Suicide |
|  | Lidocaine | 27 / M | x | Ingestion (PO) | | 1000 |  | CS | Self | Suicide |
| Spitzer 2021^33^ | Lidocaine, Ropivacaine | 52 / M |  | Block |  | 100 , 37.5 |  | CS | Anesthesia | LAST |
| Sriramatr 2021^34^ | Bupivacaine | 31 / F |  | Spinal |  | 12 |  | RR | Anesthesia | High Spinal |
|  | Bupivacaine | 80 / M |  | Spinal |  | 15 |  | RR | Anesthesia | High Spinal |
| Sud 2018^35^ | Bupivacaine | 30 / F |  | IV |  |  |  | CR |  | LAST |
| Szadkowski 2017^36^ | Lidocaine | 15 / F | x | Ingestion (PO) | | 2000 |  | CR | Self | LAST |
| Tellez-Pena 2022^37^ | Lidocaine | 60 / M |  | Block | 58 | 800 | Yes | CR | Anesthesia | LAST |
| Vadi 2014^38^ | Bupivacaine, Mepivacaine | 88 / F |  | Block | 45 | 25, 225 | Yes | CR | Anesthesia | LAST |
| Van Zyl 2017^39^ | Bupivacaine | / F |  | IV |  |  | Yes | RR | Nurse | LAST |
| Weeke 2015^40^ | Lidocaine | 0 / |  | IV | 3.9 | 4296 |  | RR | ICU | LAST |
|  | Lidocaine | 0 / |  | IV | 3.4 | 4296 |  | RR | ICU | LAST |
|  | Lidocaine | 0 / |  | IV | 3.5 | 3900 |  | RR | ICU | LAST |
|  | Lidocaine | 0 / |  | IV | 4.3 | 3415 |  | RR | ICU | LAST |
|  | Lidocaine | 0 / |  | IV | 3.2 | 3470 |  | RR | ICU | LAST |
|  | Lidocaine | 0 / |  | IV | 3.2 | 3240 |  | RR | ICU | LAST |
| Winkler 2021^41^ | Ropivacaine | 62 / F |  | IV |  | 400 |  | CS | Nurse | LAST |
|  | Ropivacaine | 78 / M |  | IV |  | 400 |  | CS | Nurse | LAST |
| Winograd 2018^42^ | Lidocaine | 33 / M | x | Ingestion (PO) | |  | Yes | CR | Self | LAST |
| Xiong 2014^43^ | Lidocaine | 56 / F |  | IV |  | 50 |  | CR | Cardiologist? | LAST |

RR: retrospective review, CR: Case review, CS: case series; EB: Endobronchial, PO: Oral ; N: nasal
