# Supplementary figures and images for: Contemporary local anaesthetic-associated adverse events and mortality: a pharmacovigilance analysis of a US reporting system
Source: Br J Anaesth. 2025 Aug 27;135(4):1015–25. doi: 10.1016/j.bja.2025.06.044 (PMC12674073; doi:10.1016/j.bja.2025.06.044)

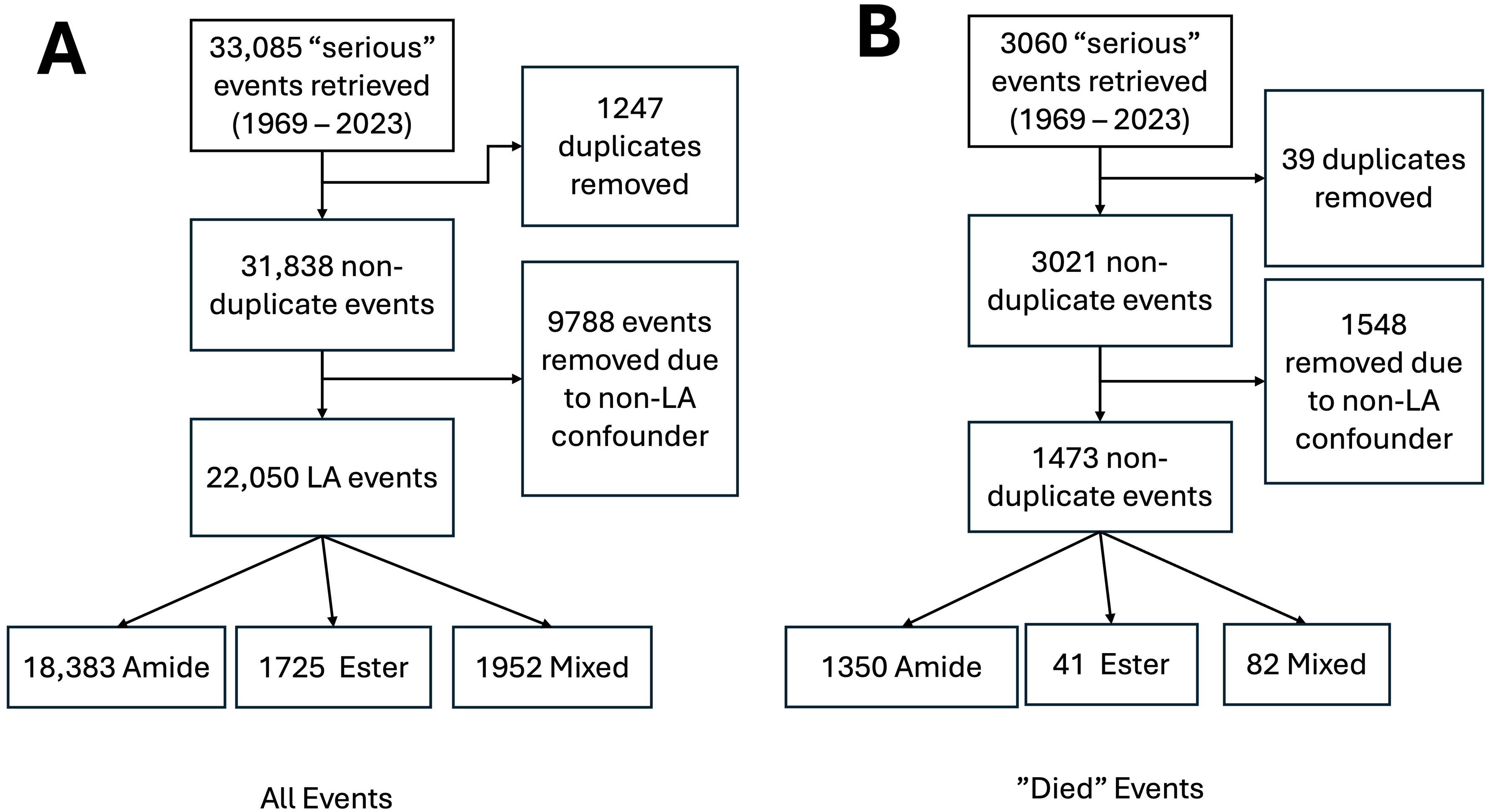

Supplement: Supplementary file 3 [file figs1.jpg]

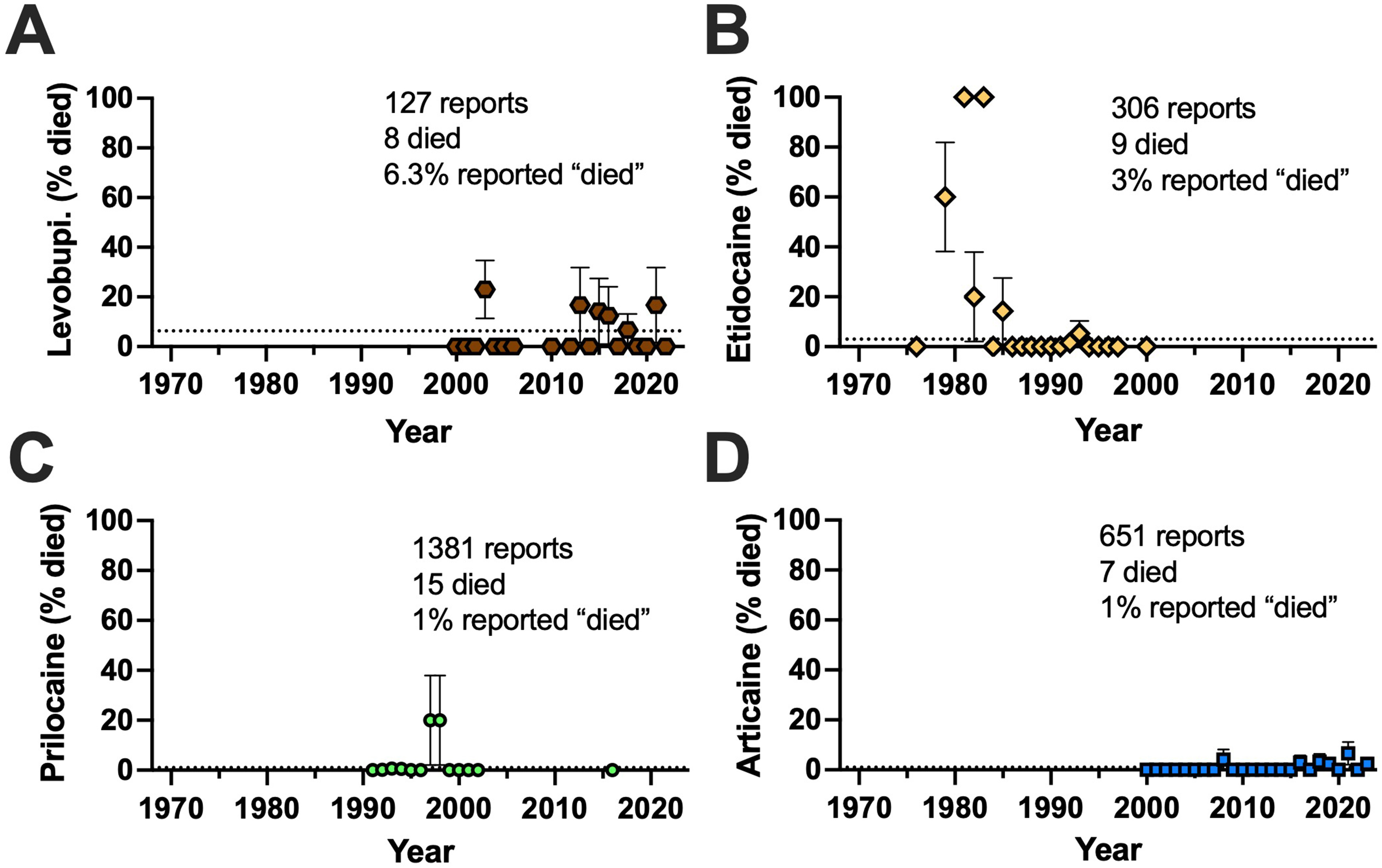

Supplement: Supplementary file 4 [file figs2.jpg]

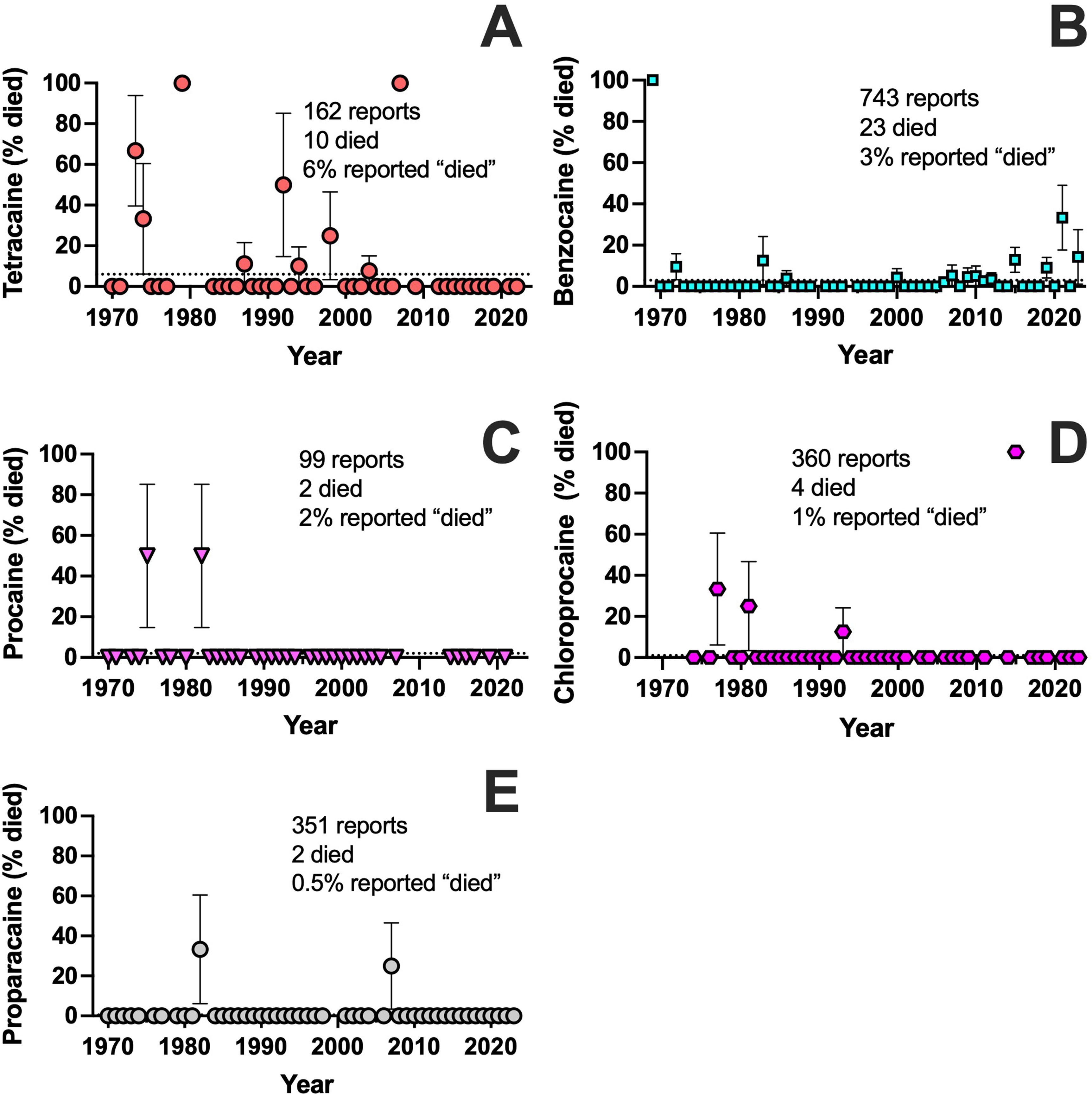

Supplement: Supplementary file 5 [file figs3.jpg]
